# Biosynthesis, Characterization, Evaluation, and Shelf-Life Study of Silver Nanoparticles against Cotton Bollworm, *Helicoverpa armigera* (Hubner) (Noctuidae: Lepidoptera)

**DOI:** 10.3390/nano12193511

**Published:** 2022-10-08

**Authors:** M.M. Anees, S.B. Patil, D.N. Kambrekar, S.S. Chandrashekhar, Shamarao Jahagirdar

**Affiliations:** Department of Agricultural Entomology, University of Agricultural Sciences, Dharwad 580005, India

**Keywords:** silver nanoparticles (AgNPs), Particle Size Analyzer, *A. indica*, *P. pinnata*, *H. armigera*

## Abstract

Nanoparticles provide a promising and alternative platform of eco-friendly technologies that encompasses better cost-resilient remedies against one of the most economically harnessing insect pests of cotton. The main goal of this research was to provide a better management strategy through biologically synthesizing (sunlight exposure method) green nanoparticles from leaf extracts of *Azadirachta indica* and *Pongamia pinnata* and proving their bioefficacy on *H. armigera* (2nd instar). Characterization of bio-synthesized silver nanoparticles was carried out using UV-Visible spectroscopy for confirming the formation of nanoparticles, a Particle Size Analyzer (PSA) for determining the size/distribution of particles, and a Scanning Electron Microscope (SEM) for analyzing the surface topology of nanoparticles. The results obtained from PSA analysis showed that *A. indica* and *P. pinnata*-based silver nanoparticles had an average diameter of 61.70 nm and 68.80, respectively. Topographical images obtained from SEM proved that most of the green synthesized silver nanoparticles were spherical in shape. *A. indica-based* silver nanoparticles were found to be comparatively more efficient and have higher insecticidal activity compared to *P. pinnata*-based nanoparticles. *A. indica*-based AgNPs recorded larval mortality of 60.00 to 93.33 percent at the concentrations of 500 to 2000 ppm, followed by *P. pinnata*-based nanoparticles, with 60.00 to 90.00 percent larval mortality. Shelf-life studies revealed that *A. indica-based* AgNPs had the maximum negative zeta potential of −58.96 mV and could be stored for three months without losing bioefficacy and up to six months with negligible reduction in bioefficacy. Symptoms caused by silver nanoparticles were leakage of body fluids, sluggishness, inactiveness, brittleness, etc.

## 1. Introduction

India is an agrarian nation with at least 80.00 percent of its population depending on agriculture for livelihood. Since pest attack is one of the major hindering factors in agriculture, different management strategies have been employed and proven to be useful for successful integrated pest management including cultural, biological, chemical tools, etc. Even though cultural, biological, and mechanical pest management strategies, etc. have been employed for integrated pest management in the early crop stages, traditionally chemical pest control is the most commonly utilized resort for managing the pest population under the threshold limit. Lack of proper knowledge about the optimum dose and luxurious application of pesticides lead to resistance development, residue deposition, pest resurgence, non-target toxicity, etc. Some of these pesticides were also found to be harmful to beneficial insects, the environment, and public health [1,2,3,4]. Feeding deterrents, fungicides, repellents, fumigants, and insecticides of biological origin have proved to be a better alternative to chemical pesticides to a limited extent. Up to now, biorational or biopesticides were the most used alternative widely employed in the field of agriculture [5].

Green nanotechnology is the branch of nanotechnology that is mainly being used for the sole purpose of enhancing environmental sustainability by minimizing the ill effects of traditional nanotechnological strategies. Nanotechnology has helped in the acting platform for revolutionizing the scenario of eco-friendly research by attaining cost-effective and easily available means in the fields of both crop production and protection. It has also offered modern and simple protocols to construct and design novel nanoparticles from single cheap sources of nanometals like silver [6], copper [7,8], and iron [9]. Composites of two or more metals like silver-copper, silver-magnesium, etc were also employed for the ecological synthesis of nanoparticles and were found to have higher antioxidant activity compared to the single metal sources [10]. Such nanoparticles are better regarded as nano-pesticides that could potentially overcome the demerits of traditional integrated pest management measures like ecological degradation, resistance development, pest resurgence, and broad toxicity of insecticides including biocontrol agents [7,11].

Nanotechnology is now found and proved to be another possible alternative to chemical pesticides. Nanoparticles are usually synthesized from biological sources like plants, fungi, bacteria [12], etc. Silver nanoparticles have been green synthesized using different plant part extracts like leaves, stem, seeds, etc. from plants with a high concentration of secondary metabolites viz., Neem (*Azadirachta indica*) [13], Soybean *(Glycine max*) [14], Tea (*Camellia sinensis*) [15], Tulsi (*Ocimum tenuiflorum*) [16], etc. External reducing and or stabilizing agents (e.g., hyaluronate biopolymer) are also employed for the biosynthesis of nanoparticles instead of plant sources [6].

Cotton bollworm, *Helicoverpa armigera* (Hub.) (Noctuidae: Lepidoptera) is one of the most influential economic pests that feed on a wide variety of agricultural crops including cotton, maize, chickpeas, and tobacco, and has been found to cause a serious amount of economic damage. In cotton crops, attacked squares open untimely/prematurely and become fruitless. Most of the damaged squares will fall off and the remaining will not produce good quality lint or produced lint will be of underling and inferior quality. The insect will also predispose the crop to infections by fungi, bacteria, and other secondary infections, ultimately leading to the rotting of squares. Direct impact on meristems of plants may inhibit development, delay maturity and result in the dropping of bolls [17,18].

The present study is carried out using silver nanoparticles synthesized from leaf extracts of *A. indica* and *Pongamia pinnata*. Different concentrations have been used against different instar larvae of *H. armigera* for finding the toxicity of nanoparticles compared to traditional chemical pesticides. Silver nanoparticles are found to act on the acetylcholinesterase enzyme that helps in neural transmission, resulting in reducing its biological activity, detoxifying enzymes, etc. Shelf-life studies were conducted to study the stability of green synthesized silver nanoparticles. Nanoparticles were stored in the room, refrigerated at freezing and deep-freezing temperatures to find out the relation between temperature and the storage life of nanoparticles.

With all the benefits in mind, nanoparticles are restricted under field conditions due to their impact on FDA (Fluorescein Diacetate) hydrolysis, antioxidants like Glutathione present in soil arthropods, and other soil parameters ultimately leading to a reduction in the microbial activity of soil and mortality of wide soil arthropod population. The lack of published materials on the impact of green nanoparticles on non-target organisms, soil structure, and microbial activity also impacts the extensification of green nanoparticles in agriculture.

## 2. Materials and Experimental Procedure

### 2.1. Materials

For the synthesis, silver nitrate powder crystals (AgNO_3_) were procured without further purification from the Nanotech laboratory, UAS Dharwad, Karnataka, India. Other components such as distilled water was used for the synthesis process and reduction of nanoparticle precursors.

#### Chemical Specifications of Silver Nitrate

Silver nitrate crystals employed as nanoparticle precursors were colorless, odorless chemicals with a density of 4.35 g/cc in the solid state. Crushed silver nitrate powder had melting and boiling points of 428.8 K and 713 K, respectively.

### 2.2. Experimental Procedure

#### 2.2.1. Insect Rearing

Laboratory culturing of cotton bollworm, *H. armigera* was initiated from the larvae collected from chickpea field (IIPR sub-research center, Dharwad, Karnataka) during the months of August–October 2019. These larvae were fed on tender leaves of cotton plants in small containers made of plastic (4.5 cm × 3.5 cm). These larvae were reared in groups from 1st to 2nd instar and exclusively (one larva per container) from the third instar onwards until they reach the pre-pupal stage. The population of pre-pupating larvae was separated from the containers and transferred carefully to the medium mixture of sand and soil for pupation. The emerged healthy adults of both sexes were then kept in clean glass chimneys covered with sterilized muslin cloth for providing a proper platform for their successful mating and oviposition. The moths were fed on sugary or 10 percent sucrose solution and settled to lay eggs on the clean muslin cloth. The cloth was then kept in a clean glass container (500 mL) over moist and clean cotton and the mouth of the container was wrapped properly with a plastic strip and a highly resistant rubber band. After the incubation period, the phototrophic larval population hatched from the eggs was carefully moved to the top of the glass container and collected with a sterilized camel hairbrush without causing any sort of contamination. These larvae were fed on tender leaves of cotton for further culturing of the population. Moreover, maintenance of larval culture (to avoid inbreeding depression) was performed by mixing adults caught at night into the laboratory population.

#### 2.2.2. Plant Extracts Preparation

Fresh and tender leaves of both *A. indica* and *P. pinnata* were collected from the medicinal plants’ field, Dept. of Horticulture, UAS Dharwad. Leaves were cleaned thoroughly with running tap water twice and followed by double distilled water to exclude all the impurities. Five grams of the weighed leaves were crushed into paste form using mortar and pestle. To the crushed leaves, 100 mL of distilled water was added and filtered with the help of filter paper (Whatman no. 1). This was then boiled at 80 °C for 60 min. This plant extract was further used for bio-synthesizing nanoparticles and kept under refrigerated conditions (4 °C) for future purposes [19].

#### 2.2.3. Green Synthesis of Silver Nanoparticles from Plant Extracts

The protocol followed by Indrakumar (2016) using sunlight exposure method was used for biosynthesis of silver nanoparticles. 10 mL of plant extracts were carefully added to 90 mL aqueous solution of precursor solution, silver nitrate (AgNO_3_-1 mM), and stored at room conditions for further use. Change in color was observed from pale yellow/light green to black/dark brown when directly exposed to sunlight for 3 h (12.00 to 3.00 pm), which confirms that the synthesis of the silver nanoparticles was successful due to the biological interaction between the metabolites present in the plant extract and silver nitrate precursor. A control of silver nitrate alone was also maintained, which did not produce any changes in color upon sunlight exposure.

#### 2.2.4. Characterization of Green Synthesized Silver Nanoparticles

Green nanoparticles were characterized using UV-visible Spectrophotometer at a wavelength ranging from 200 to 700 nm. The mean diameter and distribution of the silver nanoparticles were measured using PSA (Particle Size Analyzer (Nicomp). SEM (Scanning Electron Microscopy-Carl Zeiss-EVO-18-UK) was also used to evaluate the topology of bio-synthesized nanoparticles.

#### 2.2.5. Bioassay of Green Synthesized Silver Nanoparticles

Second instar larvae of *H. armigera* were tested for their susceptibility to different dosages of silver nanoparticles. At least five concentrations of nanoparticles (500 ppm to 2000 ppm) were treated to obtain larval mortality in the range of 10.00 to 100.00 percent. Washed and excised cotton leaf discs with a mean diameter of 50–60 mm were immersed in different concentrations of nanoparticles for 60 s. These leaf discs were then dried under shade for 60 min and kept above clean cotton tissue papers in the sterilized Petri dishes. Preliminary dosages were then determined before commencing tests for the specified treatments for establishing larval mortality in the range of 10.00 to 100.00 percent. Distilled water-treated leaf discs were served as controls for each treatment. Emamaectin benzoate at 0.30 g/L was also maintained as an insecticidal check. Ten caterpillars for *H. armigera* were provided in each replication of every treatment. Three replications of all the concentrations along with a test control were included for each nanoparticle treatment. Experimental treatments were further evaluated by observing the *H. armigera* mortality rate at treatment intervals of 24, 48, 72, and 96 h of treatment. Any larvae that could not move when disturbed on its back were considered dead [20].

Percentage reduction of *H. armigera* population over test control was performed using the formula

$$\mathrm{Percent}\;\mathrm{larval}\;\mathrm{mortality}=\frac{\mathrm{Total}\;\mathrm{number}\;\mathrm{of}\;\mathrm{dead}\;\mathrm{insects}}{\mathrm{Total}\;\mathrm{number}\;\mathrm{of}\;\mathrm{insects}\;\mathrm{treated}}\times 100$$

$$\mathrm{Corrected}\;\mathrm{percent}\;\mathrm{larval}\;\mathrm{mortality}=\frac{T-C}{100-C}\times 100\;*$$

100—C, T—Percent larval mortality in the treatment, C—Percent larval mortality in the control; * Abbott’s formulae [21].

#### 2.2.6. Shelf-Life Studies Green Synthesized Silver Nanoparticles

Shelf life of synthesized green nanoparticles against *H. armigera* and *A. devastans* was performed at the intervals of 1, 2, 3, and 6 months after the storage. Investigation on shelf life was performed at room temperature, refrigerated temperature, freezing temperature, and deep-freezing temperatures. Mortality caused by the nanoparticles at different temperatures in different intervals was calculated using Schneider Orelli’s formulae [22].

$$\mathrm{Percent}\;\mathrm{larval}\;\mathrm{mortality}\;=\frac{\mathrm{Total}\;\mathrm{number}\;\mathrm{of}\;\mathrm{dead}\;\mathrm{larvae}\;}{\mathrm{Total}\;\mathrm{number}\;\mathrm{of}\;\mathrm{larvae}\;\mathrm{treated}}\times 100$$

#### 2.2.7. Statistical Analysis

The results obtained from the study were subjected to statistical analysis (ANOVA) using a CRBD (Completely Randomized Block Design). The average values of larval mortality were then subjected to DMRT (Duncan’s Multiple Range Test).

## 3. Experimental Results

### 3.1. Biosynthesis of Silver Nanoparticles through A. indica and P. pinnata Leaf Extract

Weather parameters were not optimized for green synthesis of silver nanoparticles and the entire process was successfully performed under laboratory conditions (28–32 °C temp and 70 to 80% RH). The ratio of nanoparticle precursor solution to leaf extract solution was optimized for the successful conversion of precursor-leaf extract mixture into nanoparticles. The silver nitrate solution was mixed with *A. indica* and *P. pinnata* leaf extract solution in the ratios of 1:1.50 and 1:1.75, respectively, before exposing them to direct sunlight for nanoparticle synthesis.

### 3.2. Characterization of Silver Nanoparticles through A. indica and P. pinnata Leaf Extract

**Figure 1:**
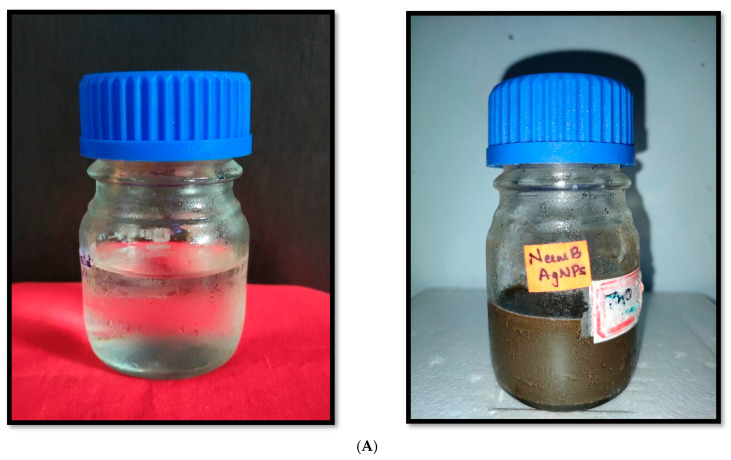

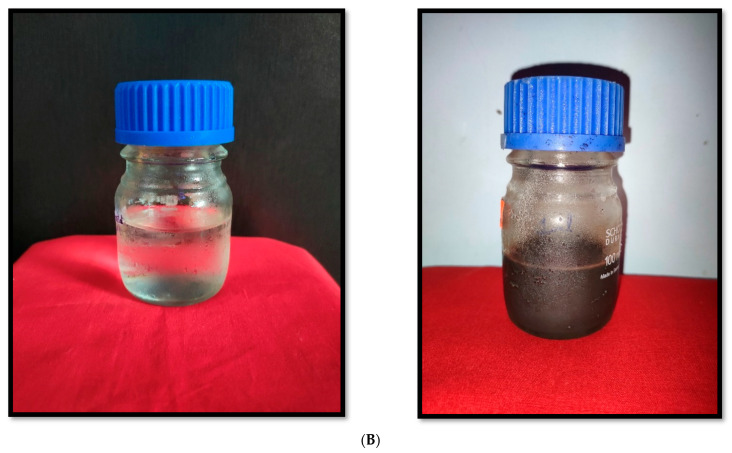
(**A**) Color variation of *A. indica*-silver nitrate solution after silver nanoparticle formation. (**B**) Color variation of *P. pinnata*-silver nitrate solution after silver nanoparticle formation.

The conversion of silver into silver ions in the green synthesized nanoparticle mixture after incubating *A. indica* and *P. pinnata* leaves extract with silver nitrate solution was positively indicated by the color change that occurred in the nanoparticle solution. Fresh suspension of silver nitrate and plant extracts were yellowish and changed to dark brownish after completion of the green synthesis process through continuous heating exposure to direct sunlight (Figure 1A,B).

**Figure 2:**
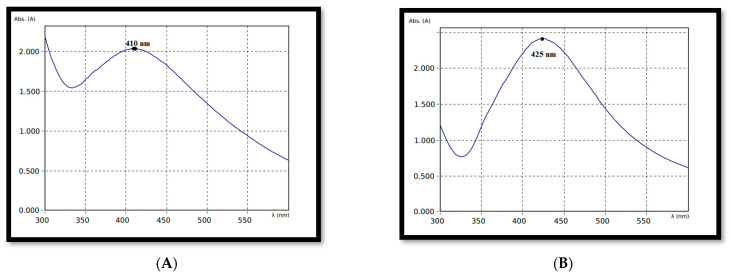
(**A**) UV visible spectroscopic image of *Azadirachta indica*-based silver nanoparticles. (**B**) UV visible spectroscopic image of *Pongamia pinnata*-based silver nanoparticles.

Confirming the formation of both *A. indica* and *P. pinnata*-based silver nanoparticles was performed using UV Spectrophotometer which produced absorbance peaks at 410 nm (Figure 2A) and 425 nm (Figure 2B), respectively. After confirming the formation of silver in the nanoparticle solution using UV Spectrophotometer, the average size of the silver nanoparticles was determined using PSA (Particle Size Analyzer-Nicomp).

**Figure 3:**
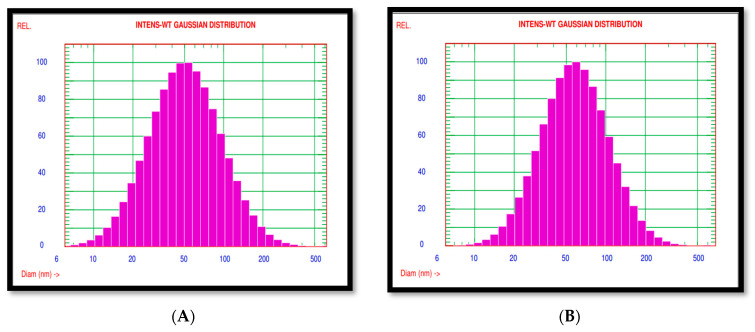
(**A**) PSAimage of *A. indica*-based AgNPs at 61.70 nm. (**B**) PSA image of *P. pinnata* AgNPs at 68.80 nm.

**Figure 4:**
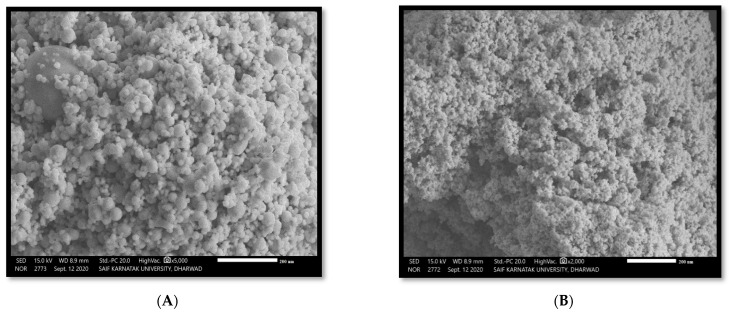
(**A**) SEM image of *A. indica*-based AgNPs at 61.70 nm. (**B**) SEM image of *P. pinnata* AgNPs at 68.80 nm.

The mean diameter of *A. indica* and *P. pinnata*-based AgNPs was 61.70 nm and 68.80 nm, respectively (Figure 3A,B). Further characterization of the nanoparticles was performed using the Surface Electron microscope. The SEM images of both nanoparticles revealed that most of the nanoparticles were mostly spherical in shape (Figure 4A,B).

### 3.3. Bio-Efficacy of Green Synthesized Silver Nanoparticles against H. armigera

Bioefficacy of *A. indica* and *P. pinnata* coated silver nanoparticles (500 to 2000 ppm) were treated on larvae of *H. armigera* (2nd instar) (Table 1 and Table 2).

#### Toxicity of Green Synthesized Silver Nanoparticles on Larval Mortality of *H. armigera*

**Figure 5:**
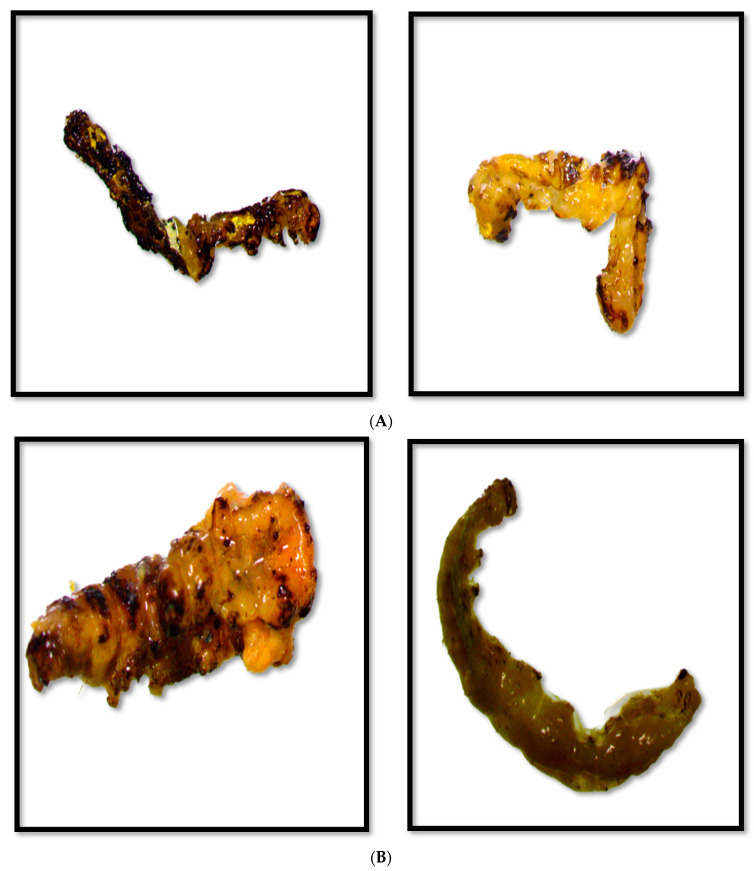
(**A**) Toxicity of *Azadirachta indica*-based AgNPs on 2nd instar larva of *Helicoverpa armigera*. (**B**) Toxicity of *Pongamia pinnata* based AgNPs on 2nd instar larva of *Helicoverpa armigera*.

The treated larva stopped feeding immediately leading to disruption in their physiology. This resulted in body rupture, leakage of body fluids, sluggishness, etc. This occurred due to various modes of action of the nanoparticles on different targets of the insect bodies. Nanoparticles physically absorbed wax and lipids from the insect cuticles after they came into contact and ultimately led to the cuticular rupture. Deformities like shrinkage, and brittleness occurred due to disruption in the nutrient uptake and altered biochemical activity. Metal nanoparticles like silver and zinc could bind to S and P present in proteins and nucleic acids, respectively, resulting in a reduction of cell membrane permeability and organelle denaturation and enzyme denaturation, and ultimately cell death. Mortality of larva was confirmed when the larvae is found unconscious and immobile when disturbed on its back or abdomen after treatment. The treated larva was found to lack body fluids, was dark brown/blackish, and hard but liable to break easily after death (Figure 5A,B).

The chemical, which was used as an insecticidal control, Emmamectin benzoate @ 0.25 g/L was found to be significantly superior to other treatments of nanoparticle treatments and recorded the highest larval mortality of 100.00 percent against 2nd instar both after 72 and 96 h of treatment. No larval mortality was recorded by a precursor (silver nitrate), the leaf extract, and double distilled water (control).

The biological efficacy of different concentrations of AgNPs against 2nd instar larvae is recorded in Table 1 and Table 2. *A. indica* and *P. pinnata*-based AgNPs recorded 10.00 to 23.33 and 10.00 to 30.00 percent larval mortality at the given concentrations (500 to 2000 ppm) after 24 h of treatment.

A similar trend in larval mortality was recorded after 48 h of treatment with larval mortality of 46.67 percent reported at 2000 ppm of both AgNPs. 1500 ppm of *A. indica*-based AgNPs registered 36.67 and 40.00 percent larval mortality by respective nanoparticle treatments. Both *A. indica* and *P. pinnata*-based AgNPs recorded mortality of 30.00 percent at 1000 ppm. Minimum larval mortality of 20.00 percent was registered by both AgNPs at 500 ppm.

At 72 h after treatment, larval mortality of 70.00 and 60.00 percent was recorded at 2000 ppm of *A. indica* AgNPs and *P. pinnata* AgNPs, respectively. Similarly, 1500 ppm of *A. indica* AgNPs recorded 60.00 percent larval mortality, followed by 1500 ppm of *P. pinnata*-based AgNPs with larval mortality of 56.67 percent. Larval mortality of 50.00 percent was registered by both AgNPs at 1000 ppm. Similarly, larval mortality of 46.67 and 40.00 percent was registered at 500 ppm of *A. indica* and *P. pinnata*-based AgNPs, respectively.

The highest larval mortality of 93.33 percent was registered at 2000 ppm of *A. indica* AgNPs, meanwhile, P. *pinnata*-based AgNPs reported 90.00 percent larval mortality after 96 h of treatment. 1500 ppm of *A. indica*-based AgNPs registered 80.00 percent larval mortality, followed. by 1000 ppm of *A. indica*-based AgNPs which reported larval mortality of 76.67 and was on par with 1500 ppm of *P. pinnata*-based AgNPs. At 500 ppm, both *A. indica* and *P. pinnata*-based AgNPs recorded the lowest larval mortality of 60.00 percent.

### 3.4. Shelf-Life Studies of Green Silver Nanoparticles against H. armigera

**Figure 6:**
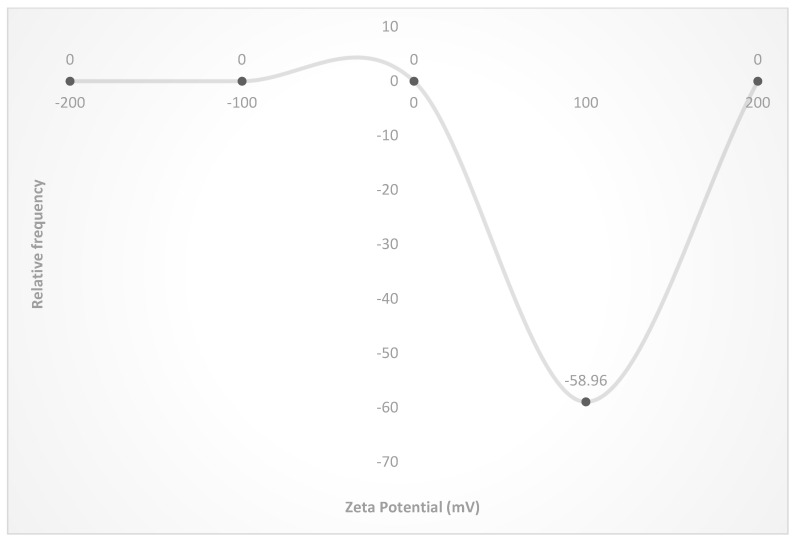
Zeta potential of *A. indica*-based AgNPs.

**Figure 7:**
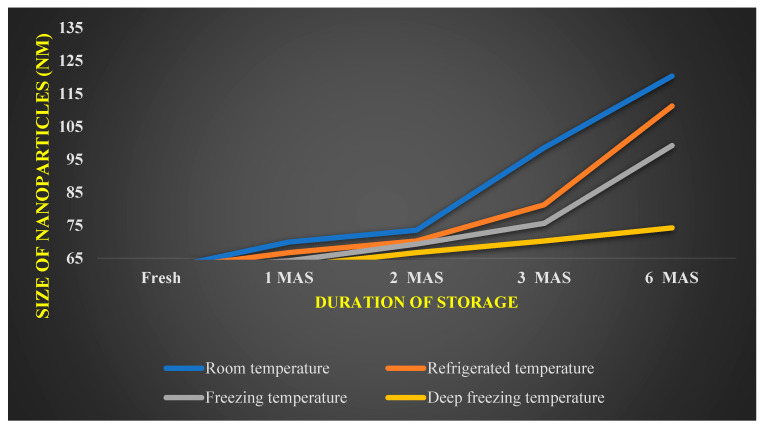
Effect of various temperatures on size of *A. indica*-based silver nanoparticles.

Zeta potential of −58.96 mV was documented by *A. indica*-based AgNPs (Figure 6 and Figure 7) showing the reduction in the size of *A. indica*-based AgNPs after one, two, three, and six months period after the storage. In AgNPs stored at room temperatures, size has increased to 69.90 nm from 61.70 nm after one month. Subsequently, size has increased to 73.50 nm, 98.6, and 120.36 nm at two, three, and six months after the storage, respectively. At refrigerated conditions, size has increased from 61.70 nm to 66.70, 70.25, 81.25, and 111.25 at one, two, three, and six months after storage, respectively. Particle size elevation to 64.2, 69.3, 75.63, and 99.25 nm was noticed at freezing temperatures after one, two, three and six months of storage, respectively. The lowest particle size elevation over storage was noticed in nanoparticles stored at deep freezing conditions. 62.50, 66.67, 70.23, and 74.23 nm were the sizes recorded at one, two, three, and six months after the storage, respectively, at deep freezing temperature. Reduction in nanoparticles’ size could be one of the factors attributed to the reduction in bioefficacy of stored nanoparticles over the freshly prepared nanoparticles.

**Figure 8:**
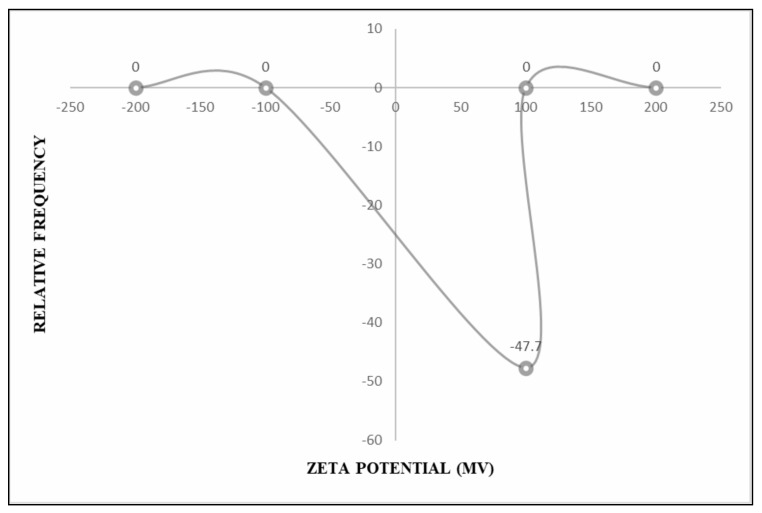
Zeta potential of *P. pinnata*-based AgNPs.

**Figure 9:**
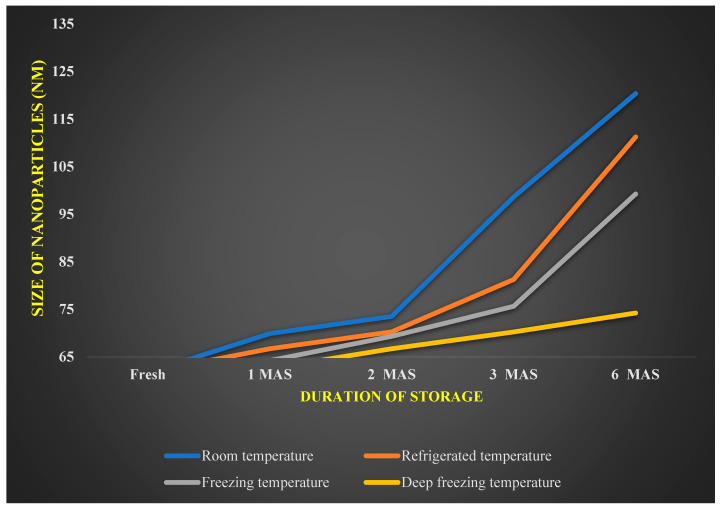
Effect of various temperatures on size of *P. pinnata*-based silver nanoparticles.

The Zeta potential of *P. pinnata* (−47.70 mV) is depicted in Figure 8, which was found to be quite low as compared to *A. indica*-based AgNPs. This implied that the stability of *P. pinnata* AgNPs was less as compared to that of *A. indica*-based AgNPs. Figure 9 conveys the increase in the size of *P. pinnata*-based AgNPs at one, two, three, and six months after storage, respectively. In AgNPs stored at room temperatures, size elevation from 68.80 nm to 72.20, 77.60, 99.60, and 135.80 nm was recorded at one, two, three, and six months after storage, respectively. AgNPs stored at room temperature showed a maximum increase in size. Refrigerated conditions followed a similar pattern with a size of 74.60, 80.23, 114.25, and 120.36 nm recorded at one, two, three, and six months after the storage, respectively. The lowest increase in particle size was noticed at freezing temperature as compared to refrigerated temperature, with a size of 70.22, 75.33, 89.22, and 100.23 nm recorded at one, two, three, and six months after storage, respectively. At the deep-freezing temperature, 70.12, 74.25, 81.32, and 96.35 nm of particles were observed at one, two, three, and six months after storage, respectively. The most ideal condition with a minimum change in the size of nanoparticles was observed when nanoparticles were stored at deep freezing temperature.

#### 3.4.1. Effect of *A. indica* Based AgNPs Stored at Different Temperatures on the Larval Mortality of *H. armigera* (Instar 1)

Larval mortality caused by AgNPs against 1st instar larvae of *H. armigera* at room, refrigerated, freezing and deep-freezing temperatures is narrated in Table 3. One month after the storage of nanoparticles, larval mortality of 50, 50, 56.67, and 70 percent was caused by AgNPs @ 500 ppm which was stored at room, refrigerated, freezing, and deep-freezing temperatures, respectively. At 1000 ppm, larval mortality of 86.67 percent was caused by AgNPs stored at deep freezing temperatures while the lowest of 60 percent was recorded by AgNPs stored at room temperature. At 1500 ppm, larval mortality of 63.33, 70.00, 76.67, and 90 percent was recorded by AgNPs stored at the room, refrigerated, freezing, and deep-freezing temperatures, respectively. Cent percent of larval mortality was registered at 2000 ppm AgNPs stored at deep freezing temperature followed by freezing, refrigerated, and room temperatures causing larval mortality of 80, 73.33, and 70 percent, respectively.

A similar trend was noticed in AgNPs stored for two months at 500 ppm where larval mortality of 40, 50, 50, and 70 percent was registered by AgNPs stored at the room, refrigerated, freezing, and deep-freezing temperatures, respectively. At 1000 ppm, 50, 56.67, 63.33, and 70 percent larval mortality was recorded by temperatures, respectively. Similarly, nanoparticles at 1500 ppm stored at room, refrigerated, freezing, and deep-freezing temperatures recorded larval mortality of 56.67, 60, 70, and 90 percent, respectively. At 2000 ppm, larval mortality of 60, 70, 80, and 100 percent were recorded at the four different temperatures, respectively.

Three months after the storage of nanoparticles, maximum larval mortality of 60, 76.67, 80, and 90 percent was recorded at 500, 1000, 1500, and 2000 ppm, respectively, by AgNPs stored at deep freezing temperature. Similarly, the lowest larval mortality of 30, 40, 40, and 50 percent was recorded at 500, 1000, 1500, and 2000 ppm of AgNPs stored at room temperature. At refrigerated temperature, 500, 1000, 1500, and 2000 ppm reported larval mortality of 40, 50, 53.33, and 60 percent, respectively. Larval mortality of 40, 56.67, 60, and 70 percent was recorded at 500, 1000, 1500, and 2000 ppm, respectively, which was stored at freezing temperature.

Six months after the storage of nanoparticles, larval mortality of 20, 20, 30, and 50 were recorded at 500, 1000, 1500, and 2000 ppm of AgNPs stored at room temperature. A similar trend was followed under both refrigerated and freezing temperatures with the highest larval mortality of 53.33 and 56.67 noticed, respectively, at 2000 ppm while minimum larval mortality of 30 percent was recorded at 500 ppm. Larval mortality of 50, 66.67, 76.67, and 80 percent recorded by 500, 1000, 1500, and 2000 ppm, respectively, was found to be superior to other treatments.

#### 3.4.2. Effect of *P. pinnata*-Based AgNPs Stored at Different Temperatures on the Larval Mortality of *H. armigera* (Instar 1)

At one month after the storage, larval mortality of 40, 50, 60, and 60 percent was caused at 500 ppm AgNPs stored in the room, refrigerated, freezing, and deep-freezing temperatures, respectively. At 1000 ppm, the highest larval mortality was caused by AgNPs stored at deep freezing temperatures (70%), and the lowest by AgNPs stored at room temperature (40%). At 1500 ppm, mortality of 46.67, 66.67, 73.33, and 73.33 percent was recorded by AgNPs stored at the room, refrigerated, freezing, and deep-freezing temperatures, respectively. Ninety percent mortality was recorded by 2000 ppm AgNPs stored at deep freezing temperature. This was followed by freezing, refrigerated, and room temperatures causing larval mortality of 80, 70, and 50 percent, respectively (Table 4).

Two months after the storage of nanoparticles, larval mortality of 30, 40, 40, and 46.67 percent was recorded by AgNPs at 500, 1000, 1500, and 2000 ppm, respectively, at room temperature. A similar trend of reduction was followed at both refrigerated and freezing temperatures with the highest larval mortality at 2000 ppm and the lowest at 500 ppm. Larval mortality of 50, 63.33, 66.67, and 90 percent was recorded at 500, 1000, 1500, and 2000 ppm, respectively, at deep freezing temperature, and was found to be superior to all other treatments (Table 4).

Three months after the storage of nanoparticles at refrigerated temperature, 500, 1000, 1500, and 2000 ppm recorded larval mortality of 30, 40, 50, and 56.67 percent, respectively. Larval mortality of 33.33, 46.67, 50, and 63.33 was recorded at 500, 1000, 1500, and 2000 ppm, respectively, at freezing temperature. Maximum larval mortality of 46.67 to 80 percent was recorded when the nanoparticles (500 to 2000 ppm) were stored at deep freezing temperature (Table 4) and were found to be superior storage condition compared to other conditions.

A similar trend was observed by AgNPs stored for six months. At 500 ppm, larval mortality of 10, 20, 30, and 40 percent was recorded by AgNPs stored in the room, refrigerated, freezing, and deep-freezing temperatures, respectively. At 1000 ppm, 20, 30, 33.33, and 50 percent larval mortality was recorded by subsequent temperatures. Similarly, nanoparticles at 1500 ppm stored at the room, refrigerated, freezing, and deep-freezing temperatures recorded larval mortality of 30, 40, 40, and 60 percent, respectively. At 2000 ppm, larval mortality of 36.67, 50, 50, and 76.67 percent was reported at the four different temperatures, respectively. Maximum and minimum larval mortality were recorded by AgNPs stored at deep-freezing and room temperatures, respectively (Table 4).

## 4. Discussion

Leaf extracts of both *A. indica* and *P. pinnata* were used for the synthesis of green AgNPs. The sunlight exposing method was successful for the synthesis and stabilization of AgNPs. The UV-Vis characteristic absorption peak of *A. indica*-based AgNPs was documented at 410 nm. The particle size was 61.70 nm and was spherical to irregular in shape. The UV-Vis characteristic absorption peak of *P. pinnata*-based AgNPs was documented at 425 nm. The particle size was 68.80 nm and was spherical to irregular in shape. Bioefficacy studies revealed that *A. indica*-based AgNPs at 2000 ppm (96 HAT) caused maximum mortality of 100.00 and 95.00 percent against 1st and 2nd instar of *H. armigera*, respectively. This was followed by *P. pinnata*-based AgNPs with 100.00 and 86.67 percent larval mortality, respectively.

An investigation conducted by Raghda et al. (2022) [23] on the larvicidal effect of green silver nanoparticles synthesized from *Artemisia herba alba* on *Spodoptera littoralis* reported that the spherical nanoparticles (9.68 to 36.7 nm) caused higher larval mortality, deformity, etc. by disintegrating the gut epithelia and deforming gonads of larvae, which is in complete agreement with the present findings.

The research findings are in full agreement with Jafir et al. (2021) [24] who reported that silver nanoparticles extracted from Tulsi, *Ocimum basilicum* caused 21.67 to 96.67 percent at concentrations of 100 to 1500 ppm against 2nd instar larva of Tobacco caterpillar, *Spodoptera litura*. Patil et al. (2016) [25] also reported similar results after evaluating silver nanoparticles of size 21–100 nm against immature stages of Tobacco caterpillar, *Spodoptera litura*.

All the findings of the present research on the implication of silver nanoparticles are in full agreement with Kasmara et al. (2018) [1] who recorded that nanoparticles produced from *Lantana camara* leaf extracts were found to be highly toxic against *Spodoptera litura* causing percent larval mortality range of 3–10 percent higher in 24 h after treatment and 13–26 percent higher in 48 h after treatment against both for 2nd and 3rd instar larvae.

Furthermore, Goutam et al. (2019) also confirmed that AgNPs were effective against all instars of *S. litura*. At 2000 ppm, larval mortality of 95.00, 80.00, 82.50, 60.00, and 60.00 percent was recorded against 1st to 5th instars of *S. litura*, respectively, by AgNPs synthesized from Soybean seed extracts after 96 h of treatment.

All the findings of the present research are in full confirmation with the study of Siva and Santhosh Kumar (2015) [26] who synthesized silver nanoparticles (Ag NPs) from leaf extracts of *Aristolochia indica* and evaluated its bioefficacy against larvae of *Helicoverpa armigera* (3rd instar). Wide range of concentrations (5 to 50 mg/mL) yielding 33.15 to 100 percent larval mortality and very effective antifeedant activity.

Further, these findings are also confirmed by the report of Durga et al. (2014) [27] who carried out a study on the toxicity of synthesized AgNPs through leaf extracts of *Euphorbia hirta* (Euphorbiaceae) against the larvae of cotton bollworm, *Helicoverpa armigera* (1st to 4th instar) and very effective mortality response to nanoparticle treatment. Larval and pupal instars showed extended durations after treatment with nanoparticles. Similar observations were also noticed in longevity and fecundity parameters.

The present investigation is also confirmed by Jyothsna and Usha (2015) [28] who reported that AgNP treatments reduced body weights in *S. litura* (3rd instar) and reduced the biological activity of detoxifying enzymes on any external agents including nanoparticles. A report of a prolonged developmental period was also noticed due to the antioxidant stress caused by AgNPs treatment.

Mojdeh et al. (2018) [29] in their research confirmed the findings and proved that nano-emulsions were highly effective against all immature stages of Mediterranean flour moth, *Ephestia kuehniella* with the highest persistence compared to other treatments including organic and inorganic products.

Similarly, these findings are strengthened by the study of Kadakaraj et al. (2018) [30] who reported the extracellular synthesis of green nanoparticles and their toxicity on larvicidalproperties against *Helicoverpa armigera*. Synthesized nanoparticles produced a larval mortality rate of 100.00 percent against both 1st and 2nd instar meanwhile 92.35 percent larval mortality was exhibited on 3rd instar.

The shelf-life studies of AgNPs and ZnNPs revealed that nanoparticles could be stored under deep freezing conditions for six months without losing much bioefficacy. Zeta potential analysis depicted that *A. indica*-based AgNps were found to be highly stable with a maximum zeta potential of −58.96 mV, followed by *P. pinnata*-based AgNPs with a zeta potential of −47.70 mV. *A. vasica* and *Asafoetida* based ZnNPs recorded zeta potential of −23.65 and −32.78 mV, respectively. AgNPs were found to be more stable with the least reduction in bio-efficacy compared to ZnNPs. Zeta potential is a correlated measure of the electrostatic repulsive force between nanoparticles. When the force between nanoparticles is not sufficient, particles show a tendency to agglomerate which results in loss of their bioefficacy due to elevation in the size. Higher zeta potential means higher electrostatic repulsive force between nanoparticles and lower probability of agglomeration. The highest zeta potential of *A. indica*-based AgNPs indicates that the electrostatic repulsive force is enough to prevent the agglomeration for up to six months. The probability of agglomeration is high in the case of *A. vasica*-based ZnNPs since the zeta potential (lower electrostatic repulsive force between nanoparticles) is found to be the lowest.

These bioefficacy results are in full agreement with the findings of Goutam et al. (2019), who reported that silver nanoparticles could be stored for up to three months under deep freezing conditions without losing their bioefficacy against *S. litura* and *Callosobruchus chinensis*. Further, results obtained by Sangeetha et al. (2014) [31] on the nanoparticles synthesis from Hing exudates exhibited stability against agglomeration for up to three months, which was in full conformity with the results obtained.

## 5. Conclusions

Direct sunlight exposure was successfully employed in the green synthesis of silver nanoparticles from leaf extracts of both *A. indica* and *P. pinnata* which acted to a greater extent as reducing, capping, and stabilizing agents. Leaf extracts converted the metallic forms of silver into cationic forms. Furthermore, the leaf extracts also acted as stabilizing agents by encapsulating the cationic forms and preventing conglomeration.

Considerable larval mortality was recorded by both *A. indica* and *P. pinnata*-based AgNPs at all four different concentrations. Both the AgNPs were found to be causing cent percent mortality at 2000 ppm after 96 h of treatment, which was on par with the insecticidal control. However, in comparison, *A. indica* AgNPs is superior since it is found to be causing higher mortality at early intervals after treatment. Higher penetration of *A. indica*-based AgNPs was attributed due to their smaller size as compared to *P. pinnata*-based AgNPs.

The shelf life studied revealed that nanoparticles could be stored for up to three months under deep freezing temperature without losing much of their efficacy. However, other storage conditions showed a considerable reduction in bio-efficacy even before three months of storage. After six months of storage, even storage conditions of deep-freezing temperature reported a reduction in bio-efficacy, but it was considerably less compared to other storage conditions.

Since the green synthesis of silver nanoparticles is comparatively easy and uses plant/ plant parts as a cheap, available and reliable source, the data obtained from probit analysis of silver nanoparticles are found to cause considerable mortality against both *H. armigera* under laboratory conditions. These results along with successful storage at deep freezing temperature proved that green nanoparticles could be put forward as an alternative for chemical pest management and a novel strategy with broad target action against pests with a different mode of habitat, habit, etc in the future. Polyhouse and field evaluation of green nanoparticles against the target pest, phytotoxic and residual studies of green nanoparticles under field conditions will further strengthen the implication of green nanoparticles in future agriculture.

## Figures and Tables

**Table 1:** Toxicity of green AgNPs. Synthesized from leaves of *Azadirachta indica* on larval mortality of *H. armigera* (Instar 2).

Concentrations	Hours After Treatment (HAT)			
	24	48	72	96
AgNPs 500 ppm	10.00 (18.43) ^d^	20.00 (26.56) ^e^	46.67 (43.09) ^e^	60.00 (50.76) ^e^
AgNPs 1000 ppm	10.00 (18.43) ^d^	30.00 (33.21) ^d^	50.00 (45.00) ^d^	76.67 (61.11) ^c^
AgNPs 1500 ppm	20.00 (21.41) ^c^	36.67 (37.27) ^c^	60.00 (50.76) ^c^	80.00 (63.43) ^c^
AgNPs 2000 ppm	23.33 (28.88) ^b^	46.67 (43.09) ^b^	70.00 (56.78) ^b^	93.33 (75.03) ^b^
AgNO_3_ (1 mM)	0.00 (0.25) ^e^	0.00 (0.25) ^f^	0.00 (0.25) ^g^	0.00 (0.25) ^g^
*A. indica* 5%	0.00 (0.25) ^e^	0.00 (0.25) ^f^	20.00 (26.56) ^f^	30.00 (33.21) ^f^
Emamectin benzoate @ 0.25 g/L	30.00 (33.21) ^a^	70.00 (56.78) ^a^	100.00 (95.00) ^a^	100.00 (95.00) ^a^
Untreated control	0.00 (0.25) ^e^	0.00 (0.25) ^f^	0.00 (0.25) ^g^	0.00 (0.25) ^g^
S.Em.±	0.78	0.98	0.68	1.30
CV	4.65	4.91	3.01	4.65
CD @ 1%	3.23	4.05	2.80	4.92

The figures in the parentheses are angular transformed values. In columns, means followed by the same letter do not differ significantly by DMRT (*p* = 0.05). AgNPs—Silver nanoparticles.

**Table 2:** Toxicity of green AgNPs synthesized from leaves of *Pongamia pinnata* on larval mortality of *H. armigera* (Instar 2).

Concentrations	Hours After Treatment (HAT)			
	24	48	72	96
AgNPs 500 ppm	10.00 (18.43) ^d^	20.00 (26.56) ^e^	40.00 (39.23) ^e^	60.00 (50.76) ^e^
AgNPs 1000 ppm	16.67 (24.10) ^c^	30.00 (33.21) ^d^	50.00 (45.00) ^d^	70.00 (56.78) ^d^
AgNPs 1500 ppm	20.00 (24.10) ^b^	40.00 (39.23) ^c^	56.67 (48.83) ^c^	76.67 (61.12) ^c^
AgNPs 2000 ppm	30.00 (26.56) ^a^	46.67 (43.09) ^b^	66.67 (54.73) ^b^	90.00 (71.56) ^b^
AgNO3 (1 mM)	0.00 (0.25) ^e^	0.00 (0.25) ^g^	0.00 (0.25) ^g^	0.00 (0.25) ^g^
*P. pinnata*-5%	0.00 (0.25) ^e^	10.00 (18.43) ^f^	20.00 (26.56) ^f^	20.00 (26.56) ^f^
Emamectin benzoate @ 0.25 g/L	30.00 (31.09) ^a^	76.67 (61.11) ^a^	100.00 (95.00) ^a^	100.00 (95.00) ^a^
Untreated control	0.00 (0.25) ^e^	0.00 (0.25) ^g^	0.00 (0.25) ^g^	0.00 (0.25) ^g^
S.Em.±	0.98	1.03	0.98	0.78
CV	4.81	4.48	4.47	2.98
CD @ 1%	3.95	4.24	4.05	3.23

The figures in the parentheses are angular transformed values. In columns, means followed by the same letter do not differ significantly by DMRT (*p* = 0.05), AgNPs—Silver nanoparticles.

**Table 3:** Effect of *A. indica*-based AgNPs stored at different temperatures on the larval mortality of *H. armigera* (Instar 1).

Concentration (ppm)	Larval Mortality at 96 HAT			
	Room Temperature (28 °C)	Refrigerated Temperature (4 °C)	Freezing Temperature (−18 °C)	Deep Freezing Temperature (−20 °C)
1 month after storage				
500	50.00 (45.00) ^e^	50.00 (45.00) ^fgh^	56.67 (48.43) ^d^	70.00 (56.78) ^f^
1000	60.00 (50.76) ^c^	66.67 (54.74) ^e^	70.00 (56.78) ^c^	86.67 (68.58) ^c^
1500	63.33 (52.73) ^b^	70.00 (56.78) ^b^	76.67 (61.12) ^b^	90.00 (71.56) ^b^
2000	70.00 (56.78) ^a^	73.33 (58.91) ^a^	80.00 (63.43) ^a^	100.00 (95.00) ^a^
2 months after storage				
500	40.00 (39.23) ^f^	50.00 (45.00) ^fg^	50.00 (45.00) ^e^	70.00 (56.78) ^f^
1000	50.00 (45.00) ^e^	56.67 (48.43) ^e^	63.33 (52.73) ^d^	83.33 (65.90) ^e^
1500	56.67 (48.83) ^d^	60.00 (50.76) ^d^	70.00 (56.78) ^c^	90.00 (71.56) ^b^
2000	60.00 (50.76) ^c^	70.00 (56.75) ^b^	80.00 (63.43) ^a^	100.00 (90.00) ^a^
3 months after storage				
500	30.00 (33.21) ^g^	40.00 (39.23) ^j^	40.00 (39.23) ^f^	60.00 (50.76) ^h^
1000	40.00 (39.23) ^f^	50.00 (45.00) ^fg^	56.67 (48.83) ^d^	76.67 (61.12) ^e^
1500	40.00 (39.23) ^f^	53.33 (46.90) ^f^	60.00 (50.76) ^d^	80.00 (63.43) ^d^
2000	50.00 (45.00) ^e^	60.00 (50.76) ^d^	70.00 (56.78) ^c^	90.00 (71.56) ^b^
6 months after storage				
500	20.00 (26.56) ^h^	30.00 (33.21) ^l^	30.00 (33.21) ^g^	50.00 (45.00) ^i^
1000	20.00 (26.56) ^h^	33.33 (35.26) ^h^	33.33 (35.26) ^h^	66.67 (54.74) ^g^
1500	30.00 (33.21) ^g^	40.00 (39.23) ^j^	40.00 (39.23) ^f^	76.67 (61.12) ^e^
2000	50.00 (45.00) ^e^	53.33 (46.90) ^i^	56.67 (48.83) ^d^	80.00 (63.43) ^d^
S.Em.±	1.41	1.22	1.52	1.72
CD @ 1%	5.49	4.76	5.92	6.66
CV (%)	5.80	4.50	5.29	4.56

Note: Figures in the parenthesis are transformed values.

**Table 4:** Effect of *P. pinnata*-based AgNPs stored at different temperatures on the larval mortality of *H. armigera* (Instar 1).

Concentration (ppm)	Larval Mortality at 96 HAT			
	Room Temperature (28 °C)	Refrigerated Temperature (4 °C)	Freezing Temperature (−18 °C)	Deep Freezing Temperature (−20 °C)
1 month after storage				
500	40.00 (39.23) ^c^	50.00 (45.00) ^e^	60.00 (50.76) ^ef^	60.00 (50.76) ^g^
1000	40.00 (39.23) ^c^	60.00 (50.76) ^c^	66.67 (54.74) ^cd^	70.00 (56.78) ^e^
1500	46.67 (43.09) ^b^	66.67 (54.74) ^b^	73.33 (58.89) ^b^	73.33 (58.89) ^d^
2000	50.00 (45.00) ^a^	70.00 (56.78) ^a^	80.00 (63.43) ^a^	90.00 (71.56) ^a^
2 months after storage				
500	30.00 (33.21) ^e^	40.00 (39.23) ^g^	46.67 (43.09) ^g^	50.00 (45.00) ^jk^
1000	40.00 (39.23) ^c^	46.67 (43.09) ^f^	50.00 (45.00) ^g^	63.33 (52.73) ^j^
1500	40.00 (39.23) ^c^	50.00 (45.00) ^e^	56.67 (48.83) ^f^	66.67 (54.74) ^f^
2000	46.67 (43.09) ^b^	60.00 (50.76) ^c^	70.00 (56.78) ^c^	90.00 (71.56) ^a^
3 months after storage				
500	20.00 (26.56) ^f^	30.00 (33.21) ^h^	33.33 (35.26) ^hi^	46.67 (43.09) ^km^
1000	30.00 (33.21) ^e^	40.00 (39.23) ^g^	46.67 (43.09) ^g^	56.67 (48.83) ^gl^
1500	36.67 (37.27) ^d^	50.00 (45.00) ^e^	50.00 (45.00) ^g^	60.00 (50.76) ^gh^
2000	40.00 (39.23) ^c^	56.67 (48.83) ^d^	63.33 (52.73) ^de^	80.00 (63.43) ^b^
6 months after storage				
500	10.00 (18.43) ^g^	20.00 (26.56) ^i^	30.00 (33.21) ^l^	40.00 (39.23) ^h^
1000	20.00 (26.56) ^f^	30.00 (33.21) ^j^	33.33 (35.26) ^hi^	50.00 (45.00) ^jkl^
1500	30.00 (33.21) ^e^	40.00 (39.23) ^g^	40.00 (39.23) ^g^	60.00 (50.76) ^g^
2000	36.67 (37.27) ^d^	50.00 (45.00) ^e^	50.00 (45.00) ^g^	76.67 (61.12) ^c^
S.Em.±	0.98	1.45	1.77	2.48
CD @ 1%	3.80	5.65	6.88	9.61
CV (%)	4.75	5.81	6.50	7.92

Note: Figures in the parenthesis are transformed values. HAT—Hours After Treatment.

## Data Availability

Not applicable.

## References

[1] Kasmara H., Melanie Nurfajri D.A., Hermawan W., Panatarani C. (2018). The toxicity evaluation of prepared *Lantana camara* nano extract against *Spodoptera litura*. (Lepidoptera: Noctuidae). AIP Conf. Proc..

[2] Okonkwo E.U., Okoye W.I. (1996). The efficacy of four seed powders and the essential oils as protectants of cowpea and maize grains against infestation by *Callosobruchus maculatus* (Fabricius) (Coleoptera: Bruchidae) and *Sitophilus zeamais* (Motschulsky) (Coleoptera: Curculionidae) in Nigeria. Int. J. Pest Manag..

[3] Subramanyam B., Hagstrum D.W. (1996). Resistance Measurement, and Management.

[4] Kamaraj C., Deepak K.P., Aiswarya D., Arul D., Amrutha V., Karthi S., Perumal P. (2018). Biopesticidal effects of *Trichoderma viridae* formulated Titanium Oxide Nanoparticles and their physiological and biochemical changes on *H. armigera*. Pestic. Biochem. Physiol..

[5] Subashini H.D., Malarvannan S., Pillai R.R. (2004). *Dodonaea angustifolia*-a potential biopesticide against *Helicoverpa armigera*. Curr. Sci..

[6] Hong W., Guobing Z., Rony M., Wei W., Linlin X., Shaofang L., Sakil M., Huihong L. (2022). Bioreduction (Ag^+^ to Ag^0^) and stabilization of silver nanocatalyst using hyaluronate biopolymer for azo-contaminated wastewater treatment. J. Alloys Compd..

[7] Benelli G., Lukehart C.N. (2017). Application of green synthesized nanoparticles in pharmacology, parasitology, and entomology. J. Clust. Sci..

[8] Ortigosa M.S., Valenstein J.S., Lin VS Y., Trewyn BGWang K. (2012). Gold functionalized mesoporous silica nanoparticles mediated protein and DNA codelivery to plant cells via the holistic method. Adv. Funct. Mater..

[9] Waqas A., Arif U.K., Saira S., Lei Qin Qipeng Y., Aftab A., Yun W., Zia U.H.K., Sadeeq U., Aziz U.R. (2020). Eco-benign approach to synthesize spherical iron oxide nanoparticles: A new insight in photocatalytic and biomedical applications. J. Photochem. Photobiol. B Biol..

[10] Afaq U.K., Arif U.K., Baoshan L., Mater H.M., Bandar A.A., Yahya S.A., Ali O.A., Zia U.H.K., Sami U., Muhammad W. (2021). Biosynthesis of silver capped magnesium oxide nanocomposite using *Olea cuspidata* leaf extract and their photocatalytic, antioxidant and antibacterial activity. Photodiagn. Photodyn. Ther..

[11] Smith K., Evans D.A., Hiti G.A.E. (2008). Role of modern chemistry in sustainable arable crop protection. Philos. Trans. R. Soci..

[12] Zia H.K., Noor S.S., Jibran I., Arif U.K., Muhammad I., Saad M., Alshehri Nawshad M.S., Naveed A., Amina K., Munazza A. (2020). Biomedical and photocatalytic applications of biosynthesized silver nanoparticles: Ecotoxicology study of brilliant green dye and its mechanistic degradation pathways. J. Mol. Liq..

[13] Tripathi A., Chandrasekaran N., Raichur A.M., Mukherjee A. (2009). Antibacterial applications of silver nanoparticles synthesized by aqueous extract of *Azadirachta indica* leaves. J. Biomed. Nanotechnol..

[14] Vivekanandhan S., Misra M., Mohanty A.K. (2009). Biological synthesis of silver nanoparticles using *Glycine max* (soybean) leaf extract; an investigation on different Soybean varieties. J. Nanosci. Nanotechnol..

[15] Begum N.A., Mondal S., Basu S., Laskar R.A. (2009). Biogenic synthesis of Au and Ag nanoparticles using aqueous solutions of black tea leaf extracts. Col. Surf. Biointer..

[16] Rony M., Salauddin M.S.K., Zubair B.S.O., Taosif A., Shabrina K., Azhar M.W. (2021). Functionalizing cotton fabrics through herbally synthesized nanosilver. Clean. Eng. Technol..

[17] Basu A.K., Sundarmurthy V.T.A.K. (1985). Management of cotton insect pests in poly crop system in India. Outlook Agric..

[18] Manjunath T.M., Bhatnagar V.S., Pawar C.S., Sithanantham S. (1989). Economic importance of *Heliothis* spp. in India and an assessment of their natural enemies and host plants. Proceedings of the Workshop on Biological Control of Heliothis: Increasing the Effectiveness of Natural Enemies.

[19] Indrakumar N. (2016). Effect of Green Nanoparticles on Spodoptera Litura and Bombyx Mori. Master’s Thesis.

[20] Goutam B.H., Patil R.R., Benagi V.I., Chandrashekhar S.S., Nandihali B.S. (2019). Synthesis of Green Silver Nanoparticles from Soybean Seed and its Bioefficacy on *Spodoptera litura* (F.). Int. J. Curr. Microbiol. App. Sci..

[21] Abbott W.S. (1925). A method for computing the effectiveness of an insecticide. J. Econ. Entomol..

[22] Püntener W. (1981). Manual for Field Trials in Plant Protection.

[23] Raghda S.E., Magda H.R., Bouthiana A.M., El-sheikh T.A.A., Rasha E.H., El G. (2022). Larvicidal and pathological effects of green synthesized silver nanoparticles from *Artemisia herba-alba* against *Spodoptera littoralis* through feeding and contact application. Egypt. J. Basic Appl. Sci..

[24] Jafir M., Jam N.A., Muhammed J.A., Safdar A., Samina J.N.A. (2021). Characterization of *Ocimum basilicum* synthesized silver nanoparticles and its relative toxicity to some insecticides against tobacco cutworm, *Spodoptera litura* Fab. (Lepidoptera; Noctuidae). Ecotoxicol. Environ. Saf..

[25] Patil R.R., Nargund V.B., Hasansab A.N., Puneeth Raj M.S., Indrakumar N., Chikkanna S., Taranath T.C. Evaluation of synthesized green nanoparticles with plant extracts against *Tobacco caterpillar*, *Spodoptera litura* (Fab.). Proceedings of the International Conference on Nanotechnology (ICNANO).

[26] Siva C., Santhosh K.M. (2015). Pesticidal activity of eco-friendly synthesized silver nanoparticles using *Aristolochia indica* extract against *Helicoverpa armigera* Hubner (Lepidoptera: Noctuidae). Int. J. Adv. Sci. Tech..

[27] Durga G.D., Murugan K., Selvam C.P. (2014). Green synthesis of silver nanoparticles using *Euphorbia hirta* (Euphorbiaceae) leaf extract against crop pest of cotton bollworm (Lepidoptera: Noctuidae). J. Biop..

[28] Jyothsna Y., Usha R.P. (2015). Lepidopteran insect susceptibility to silver nanoparticles and measurement of changes in their growth, development, and physiology. Chemosphere.

[29] Mojdeh L., Jahansir S., Maryam N. (2018). Insecticidal efficacy of nanoemulsion containing Mentha longifolia oil against *Ephestia kuehniella* (Lepidoptera: Pyralidae). J. Crop Prot..

[30] Murugan K., Roni M., Panneerselvam C., Suresh U., Rajaganesh R., Aruliah R., Mahyoub J.A., Trivedi S., Rehman H., Al-Aoh H.A.N. (2018). *Sargassum wightii*-synthesized ZnO nanoparticles reduce the fitness and reproduction. of the malaria vector *Anopheles stephensi* and cotton bollworm *Helicoverpa armigera*. Physiol. Mol. Plant Pathol..

[31] Sangeetha J., Sandhya J., Philip J. (2014). Biosynthesis and functionalization of silver nanoparticles using *Nigella sativa*, *Dioscorea alata* and *Ferula asafoetida*. Sci. Adv. Mater..

